# MicroRNA-668-3p inhibits myoblast proliferation and differentiation by targeting Appl1

**DOI:** 10.1186/s12864-023-09431-0

**Published:** 2023-07-24

**Authors:** Haigang Cao, Tianning Du, Chenchen Li, Lingling Wu, Jieming Liu, Yuan Guo, Xiao Li, Gongshe Yang, Jianjun Jin, Xin’e Shi

**Affiliations:** 1 Laboratory of Animal Fat Deposition and Muscle Development, Key Laboratory of Animal Genetics, Breeding and Reproduction of Shaanxi Province, College of Animal Science and Technology, Northwest A&F University, Yangling, Shaanxi China; 2 Microbial Research Institute of Liaoning Province, Chaoyang, Liaoning China

**Keywords:** miR-668-3p, Cell proliferation, Cell differentiation, Myogenesis, Appl1

## Abstract

**Background:**

Skeletal muscle is the largest tissue in the body, and it affects motion, metabolism and homeostasis. Skeletal muscle development comprises myoblast proliferation, fusion and differentiation to form myotubes, which subsequently form mature muscle fibres. This process is strictly regulated by a series of molecular networks. Increasing evidence has shown that noncoding RNAs, especially microRNAs (miRNAs), play vital roles in regulating skeletal muscle growth. Here, we showed that miR-668-3p is highly expressed in skeletal muscle.

**Methods:**

Proliferating and differentiated C2C12 cells were transfected with miR-668-3p mimics and/or inhibitor, and the mRNA and protein levels of its target gene were evaluated by RT‒qPCR and Western blotting analysis. The targeting of Appl1 by miR-668-3p was confirmed by dual luciferase assay. The interdependence of miR-668-3p and Appl1 was verified by cotransfection of C2C12 cells.

**Results:**

Our data reveal that miR-668-3p can inhibit myoblast proliferation and myogenic differentiation. Phosphotyrosine interacting with PH domain and leucine zipper 1 (Appl1) is a target gene of miR-668-3p, and it can promote myoblast proliferation and differentiation by activating the p38 MAPK pathway. Furthermore, the inhibitory effect of miR-668-3p on myoblast cell proliferation and myogenic differentiation could be rescued by Appl1.

**Conclusion:**

Our results indicate a new mechanism by which the miR-668-3p/Appl1/p38 MAPK pathway regulates skeletal muscle development.

**Supplementary Information:**

The online version contains supplementary material available at 10.1186/s12864-023-09431-0.

## Background

Skeletal muscle is the most abundant and highly heterogeneous tissue in the body, skeletal muscle is characterized by strong contraction and high plasticity, and it plays a crucial role in motion, metabolism and homeostasis [1, 2]. Skeletal muscle development mainly includes four stages: terminal differentiation of mesenchymal stem cells into myoblasts, proliferation and differentiation of myoblasts into primary myotubes, maturation of myotubes into myofibers, and myofiber hypertrophy [3]. Myogenesis is a rigorous and orderly biological process, that is strictly regulated by members of the myogenic regulatory factor (MRF) family, including myogenic differentiation 1 (MyoD), myogenin (MyoG), myogenic factor 5 (Myf5), and muscle-specific regulatory factor 4 (MRF4, also known as Myf6), as well as members of the myocyte enhancer factor 2 (MEF2) family [4–6]. The precise regulation of the spatiotemporal expression of these transcription factors is essential for muscle growth. For instance, epigenetic regulatory mechanisms, particularly the histone modifications H3K27me3 and K3K4me3, are dynamically altered during myogenesis, which is required for the proper expression of myogenic genes and myoblast differentiation [7]. In addition, myogenesis is also regulated by multiple signalling pathways [8]; for example, the p38 mitogen-activated protein kinase (MAPK) pathway can activate the transcriptional activity of MRFs and MEF2, which is necessary for myoblast differentiation [9, 10].

In recent years, an increasing number of studies have shown that noncoding RNAs, especially microRNAs (miRNAs), also play important roles in muscle development [11, 12]. MiRNAs are a class of endogenous noncoding, small, single-stranded RNAs with a length of approximately 22 nucleotides; these molecules are widely expressed throughout the body and are highly conserved [13]. Routke et al. specifically knocked out the Dicer enzyme (an enzyme that is essential for miRNA maturation) in the skeletal muscle of mice and observed perinatal death, decreased skeletal muscle mass, and abnormal muscle fibre morphology in these mice, suggesting that miRNAs are key components that are necessary for skeletal muscle development [14]. According to the expression profiles of miRNAs in muscle, miRNAs can be divided into two types: the first type is miRNAs that are specifically expressed in muscle, called MyomiRs, such as miR-1, miR-133, miR-206, miR-208, miR-486 and miR-499 [15]; the other type is miRNAs that are widely expressed in tissues, such as miR-181, miR-125b, and miR-155, and play important roles in skeletal muscle development [16–18]. miR-668-3p is widely expressed in tissues. Previous studies have shown that miR-668-3p enhances radioresistance in breast cancer cells [19], maintains renal tubular cell survival [20], inhibits neuronal apoptosis during cerebral ischaemic stroke [21], reduces ischaemic apoptosis in cardiac myocytes [22], regulates hepatocellular carcinoma tumorigenesis [23], and serves as a marker of tissue hypoxia after traumatic haemorrhagic shock [24]. These studies reveal that miR-668-3p plays important roles in disease; however, the function of miR-668-3p in skeletal muscle is unclear.

An adaptor protein that contains a PH domain, PTB domain and leucine zipper motif 1 (Appl1) was the first molecule that was shown to mediate adiponectin function, and it positively mediates adiponectin signalling to the Adenosine 5‘-monophosphate (AMP)-activated protein kinase (AMPK) and p38 MAPK pathways, leading to increased glucose uptake and fatty acid oxidation in myoblasts [25]. In addition, Appl1 can also regulate skeletal muscle glucose and fatty acid metabolism, insulin resistance and myogenic differentiation by regulating the phosphatidylinositol-3-kinase-serine/threonine kinase (PI3K-AKT) signalling pathway [26]. These results suggest that Appl1 is an important regulator of muscle homeostasis and myogenic differentiation.

In this study, we found that miR-668-3p is highly expressed in mouse skeletal muscle and that its expression level is altered during myoblast proliferation and differentiation. Overexpression of miR-668-3p inhibits myoblast proliferation and myogenic differentiation, while knockdown of miR-668-3p promotes myoblast proliferation and myogenic differentiation. Using bioinformatics analysis and dual luciferase reporter assays, we identified Appl1 as a target gene of miR-668-3p that promotes myoblast proliferation and differentiation. Furthermore, we found that miR-668-3p inhibits myoblast proliferation and differentiation mainly by targeting Appl1 and inhibiting the p38 MAPK signalling pathway. In summary, our study reveals the important role of the miR-668-3p/Appl1/p38 MAPK signalling axis in myogenesis.

## Results

### MiR-668-3p was highly expressed in skeletal muscle

**Fig. 1:**
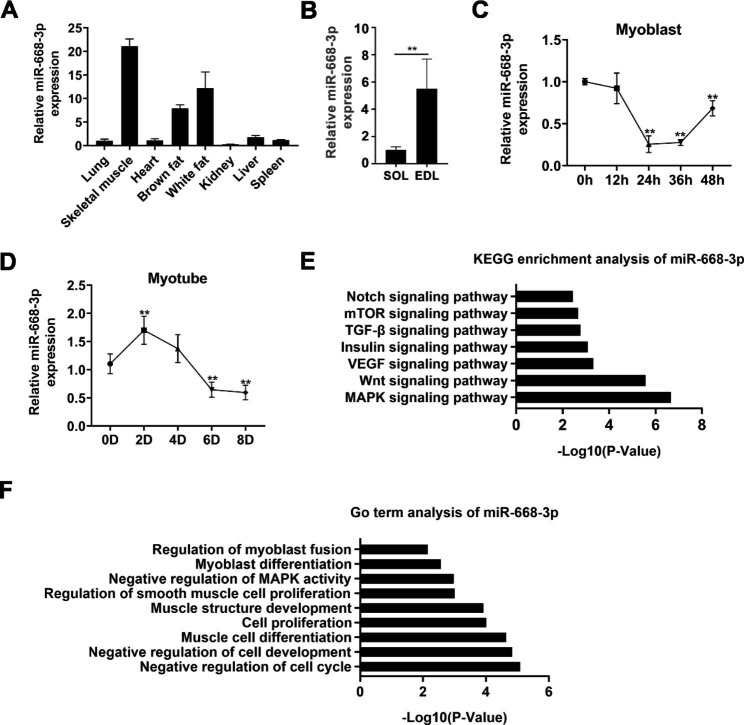
The expression pattern of miR-668-3p. **A** Relative expression level of miR-668-3p in 5-week-old mouse tissues. **B** Relative expression level of miR-668-3p in the soleus (Sol) and extensor digitorum longus (EDL) muscles. **C** The expression levels of miR-668-3p at 0, 12, 24, 36 and 48 h of cell proliferation. **D** The expression levels of miR-668-3p at 0, 2, 4, 6 and 8 days after myoblast differentiation was induced. U6 was used as the reference gene. **E** KEGG pathway analysis of the miR-668-3p target genes. **F** GO term analysis of the miR-668-3p target genes. The data represent the mean ± SD from at least three independent experiments. (**, *P* < 0.01)

To determine the expression pattern of miR-668-3p in skeletal muscle and myocytes, we measured the expression level of miR-668-3p in the tissues of 5-week-old mice, and the results showed that miR-668-3p was predominantly expressed in leg muscle and adipose tissue (Fig. 1A). Additionally, we measured the expression of miR-668-3p in different muscle fibre types, and it was highly expressed in the extensor digitorum longus (EDL) muscle (Fig. 1B). In addition, we found that miR-668-3p was highly expressed during the early proliferation of myoblast cells, and its expression gradually decreased during the proliferation process (Fig. 1C). Similarly, the expression of miR-668-3p was downregulated in terminally differentiated myotubes (Fig. 1D). We further performed GO term analysis on miR-668-3p target genes and found that miR-668-3p may be involved in muscle development, especially muscle cell proliferation and differentiation (Fig. 1F). Moreover, KEGG pathway analysis revealed that miR-668-3p may regulate biological processes through the MAPK signalling pathway (Fig. 1E). Together, our results indicated that miR-668-3p is a potential regulator of myogenesis.

### MiR-668-3p inhibits myoblast proliferation

**Fig. 2:**
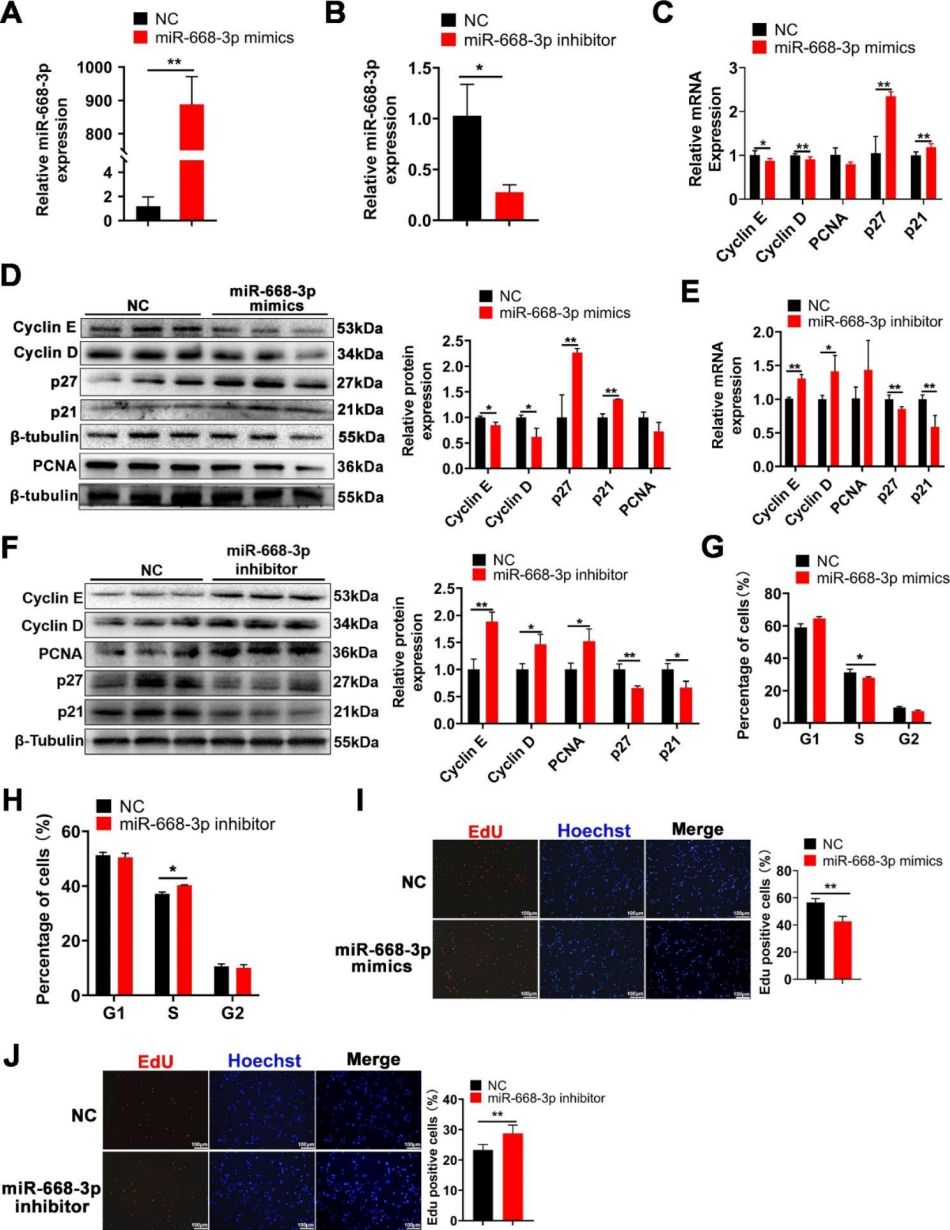
miR-668-3p inhibits myoblast proliferation. **A**, **B** The efficiency of miR-668-3p overexpression and interference. **C**, **E** The mRNA expression of Cyclin E, Cyclin D PCNA, p27 and p21 expression after miR-668-3p mimic or inhibitor transfection was measured using RT‒qPCR. **D**, **F** The protein expression of Cyclin E, Cyclin D, PCNA, p27 and p21 was determined using Western blotting and quantified by ImageJ. The membrane was cleaved prior to hybridization with the antibody, and all protein bands in Figure D and protein bands in Figure F were from the same sample, respectively. Full-length blots/gels are presented in Supplementary Fig. S1. **G**, **H** Cell cycle analysis of myoblasts after treatment with miR-668-3p mimics or inhibitor by flow cytometry and statistical analysis of the flow cytometry results. **I**, **J** EdU staining assay after treatment with miR-668-3p mimics or inhibitor. Myoblasts in the S phase were stained with EdU in red, and cell nuclei were stained with Hoechst in blue. Quantification ratio of EdU-positive cells/total cells. The data represent the mean ± SD from at least three independent experiments. (*, *P* < 0.05; **, *P* < 0.01)

To explore the role of miR-668-3p in myogenesis, miR-668-3p was overexpressed and knocked down in C2C12 cells (Fig. 2A, B). Overexpression of miR-668-3p significantly inhibited the expression of the proliferation-related genes Cyclin E, Cyclin D and PCNA, promoted p27 and p21 expression at the mRNA and protein levels (Fig. 2C, D). However, miR-668-3p knockdown had the opposite effects (Fig. 2E, F). Additionally, flow cytometry assays showed a significant decrease and increase in DNA replication (S-phase) after miR-668-3p overexpression and knockdown, respectively (Fig. 2G, H). Furthermore, EdU staining showed that miR-668-3p significantly decreased the number of EdU-positive cells (Fig. 2I, J). These results indicate that miR-668-3p inhibits myoblast proliferation.

### MiR-668-3p inhibits myogenic differentiation

**Fig. 3:**
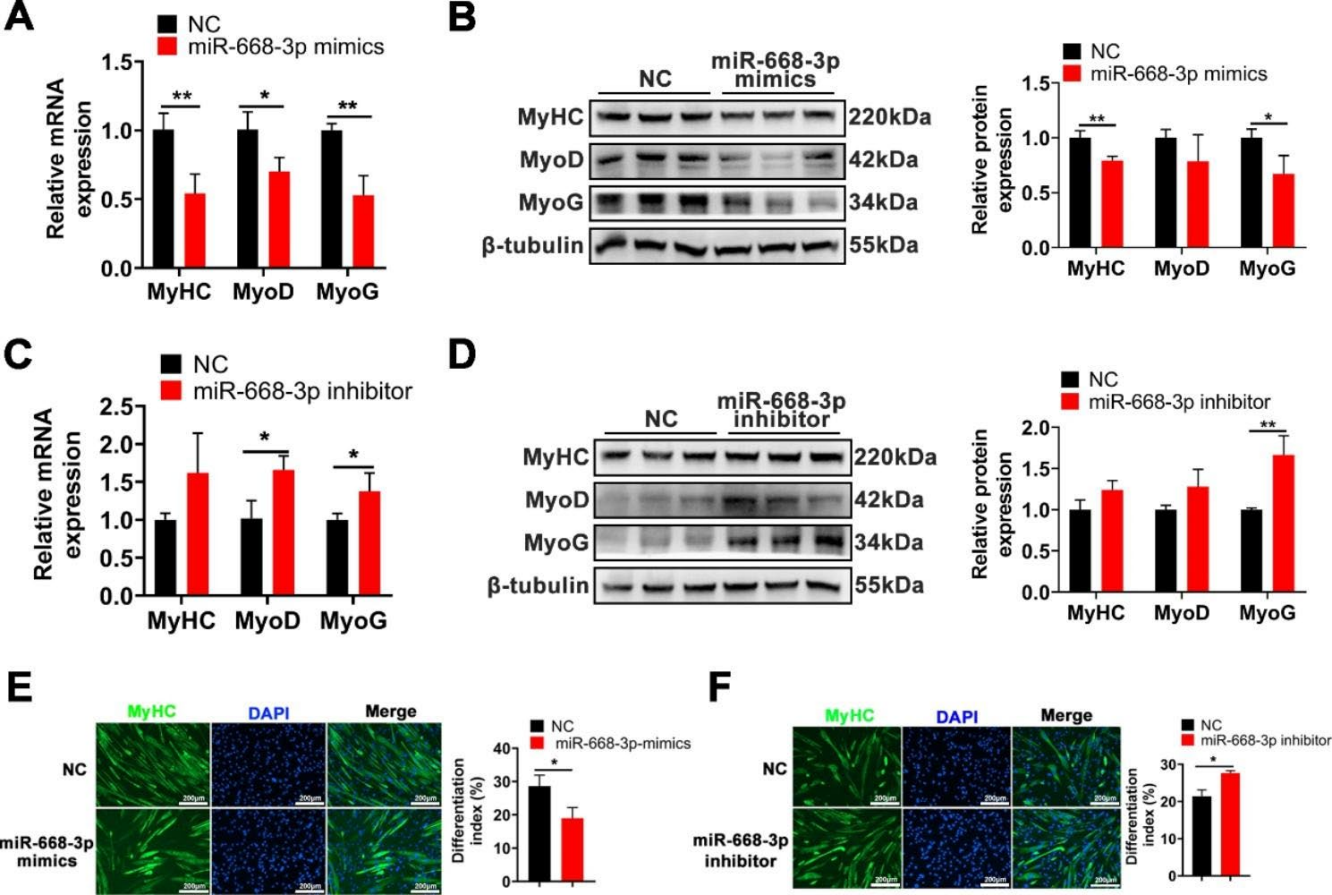
miR-668-3p inhibits myogenic differentiation. **A**, **C** The mRNA levels of MyHC, MyoD and MyoG were determined after overexpression and knockdown of miR-668-3p. **B**, **D** The protein levels of MyHC, MyoD, and MyoG after treatment with miR-668-3p mimics or inhibitor were measured by Western blotting and quantified by ImageJ. The membrane was cleaved prior to hybridization with the antibody, and all protein bands in Figure B and protein bands in Figure D were from the same sample, respectively. Full-length blots/gels are presented in Supplementary Fig. S2. **E**, **F** MyHC immunofluorescence staining and differentiation index after overexpression and knockdown of miR-668-3p. The data represent the mean ± SD from at least three independent experiments. (*, *P* < 0.05; **, *P* < 0.01)

To elucidate the effect of miR-668-3p on myogenic differentiation, myoblasts were transfected with miR-668-3p mimics or inhibitor, and then differentiation was induced. RT‒qPCR and Western blotting assays showed that overexpression of miR-668-3p significantly inhibited the expression of myogenic genes, including MyoD, MyoG, and MyHC, at the mRNA and protein levels (Fig. 3A, B). Conversely, inhibition of miR-668-3p promoted myogenic gene expression (Fig. 3C, D). Furthermore, immunofluorescence staining of MyHC showed that overexpression of miR-668-3p inhibited myotube formation and reduced myogenic differentiation (Fig. 3E), while inhibition of miR-668-3p promoted myogenic differentiation (Fig. 3F). Together, these results demonstrated that miR-668-3p inhibits myoblast differentiation.

### MiR-668-3p directly targets Appl1

**Fig. 4:**
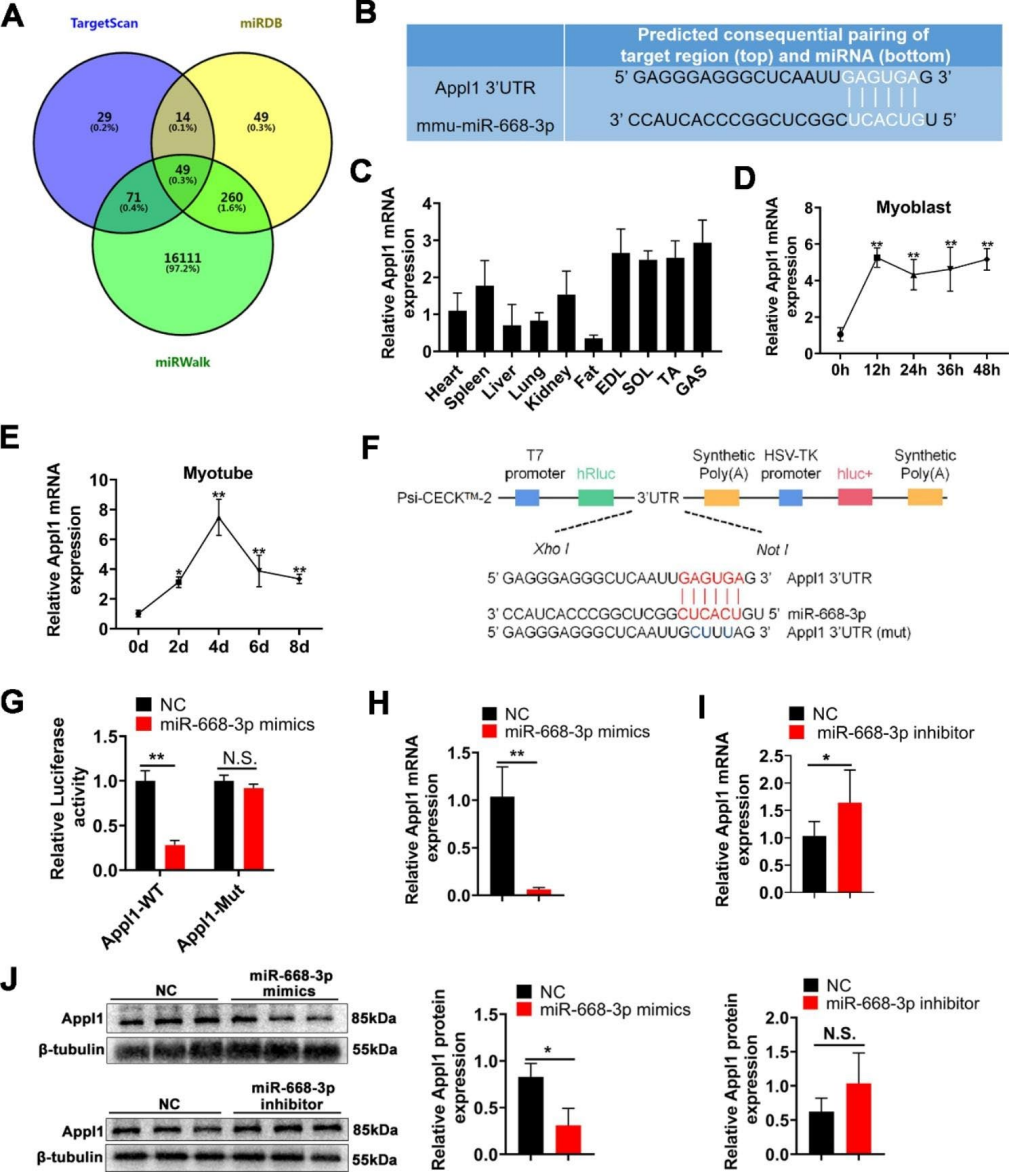
miR-668-3p directly targets Appl1. **A** Venn diagram of predicted target genes of miR-668-3p. **B** Prediction of the binding site of miR-668-3p in the Appl1 3’UTR. **C** Relative expression levels of Appl1 in 5-week-old mouse tissues. **D** The mRNA levels of Appl1 at 0, 12, 24, 36, and 48 h of cell proliferation. The fold change is shown relative to Day 0 of GM expression. **E** The mRNA levels of Appl1 at 0, 2, 4, 6 and 8 days of cell differentiation. **F** Construction of the dual-luciferase reporter psiCHECK-2-Appl1 3’UTR. **G** Dual-luciferase reporter assays were performed after cotransfection of miR-664-5p mimics and WT or mutant vectors. The relative luciferase activity is presented as Renilla luciferase/firefly luciferase. **H**, **I** Relative mRNA expression of Appl1 after treatment with miR-668-3p mimics and inhibitor. **J** Western blotting analysis of Appl1 protein expression after transfection with miR-664-5p mimics and inhibitor. The membrane was cleaved prior to hybridization with the antibody, and the protein bands from the same experiment in Figure J are from the same membrane. Full-length blots/gels are presented in Supplementary Fig. S3. The data represent the mean ± SD from at least three independent experiments. (*, *P* < 0.05; **, *P* < 0.01)

To understand the regulatory effect of miR-668-3p on myogenesis, we predicted the target genes of miR-668-3p using TargetScan, miRDB, and miRWalk online software, and 49 target genes were predicted by the different systems. (Fig. 4A). Among these target genes, we mainly focused on the Appl1 gene (Fig. 4B). A previous study reported that Appl1 promotes myogenic differentiation [26]. Therefore, Appl1 was used as a candidate gene for further research. We first examined the expression profiles of Appl1 in tissues and during myogenesis. The results showed that Appl1 was highly expressed in skeletal muscle, indicating that Appl1 was involved in the development of skeletal muscle (Fig. 4C). Furthermore, the expression levels of *Appl1* gradually increased in C2C12 cells from Day 0 (proliferating myoblasts) to Day 4 of differentiated myotubes, and then decreased (Fig. 4D, E), which was opposite of the expression pattern of miR-668-3p.

We next confirmed the effect of miR-668-3p on the expression level of the Appl1 gene. The dual-luciferase reporter assays showed that miR-668-3p mimics markedly inhibited the luciferase activity of the wild-type psiCHECK-2-Appl1 3’UTR reporter; however, the dual fluorescence activity of the vector carrying the mutated miR-668-3p binding site did not significantly change (Fig. 4F, G). As expected, overexpression of miR-668-3p significantly inhibited the mRNA and protein expression of Appl1 (Fig. 4H, J). However, miR-668-3p knockdown significantly increased only the mRNA expression of *Appl1*, and the change in its protein expression was not significant (Fig. 4I, J). All of these results demonstrated that Appl1 is a direct target gene of miR-668-3p.

### Appl1 promotes myoblast proliferation and differentiation

**Fig. 5:**
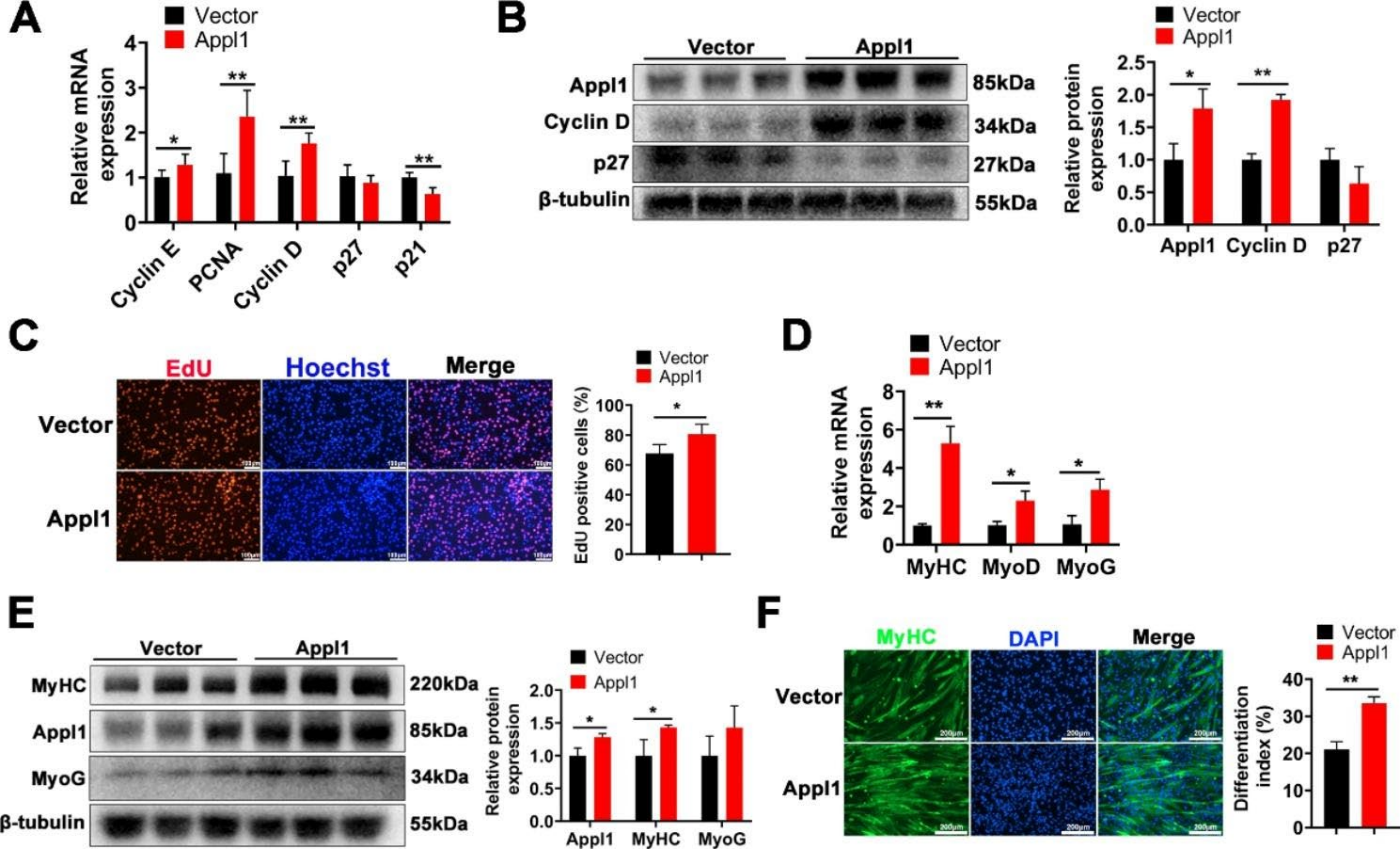
Appl1 promotes myoblast proliferation and differentiation. **A** The mRNA expression of Cyclin E, PCNA, Cyclin D, p21 and p27 at 24 h after overexpression of Appl1 was measured using RT‒qPCR. **B** The protein expression of Appl1, cyclin D and p27 was determined using Western blotting and quantified by ImageJ. The membrane was cleaved prior to hybridization with the antibody, and the protein bands in Figure B are from the same membrane. Full-length blots/gels are presented in Supplementary Fig. S4A. **C** EdU staining after transfection with the Appl1 overexpression vector. Quantification the ratio of EdU-positive cells/total cells. **D** The mRNA expression of MyHC, MyoD and MyoG after overexpression of Appl1 was measured using RT‒qPCR. **E** The protein expression of Appl1, MyHC and MyoG was determined using Western blotting and quantified by ImageJ. The membrane was cleaved prior to hybridization with the antibody, and the protein bands in Figure E are from the same membrane. Full-length blots/gels are presented in Supplementary Fig. S4B. **F** MyHC immunofluorescence staining and differentiation index after Appl1 overexpression. The data represent the mean ± SD from at least three independent experiments. (*, *P* < 0.05; **, *P* < 0.01)

To elucidate the role of Appl1 in myogenesis, we overexpressed Appl1 in C2C12 cells. The results showed that Appl1 significantly increased the mRNA levels of the proliferation-promoting genes *Cyclin E*, *PCNA*, and *Cyclin D* and decreased the mRNA levels of the proliferation-suppressing gene *p21* (Fig. 5A). In addition, Appl1 significantly upregulated the protein expression of Cyclin D and downregulated the protein expression of p27 (Fig. 5B). EdU staining revealed that Appl1 significantly increased the proportion of EdU-positive cells, suggesting that Appl1 promotes myoblast proliferation (Fig. 5C). Furthermore, overexpression of Appl1 significantly increased the mRNA (Fig. 5D) and protein (Fig. 5E) expression of the myogenic genes MyHC, MyoD and MyoG. Immunofluorescence staining of MyHC showed that Appl1 overexpression significantly increased the number of myotubes (Fig. 5F). The above results show that Appl1 could promote myoblast proliferation and myogenic differentiation.

### MiR-668-3p inhibits myogenesis mainly by targeting Appl1 and inhibiting MAPK signalling

**Fig. 6:**
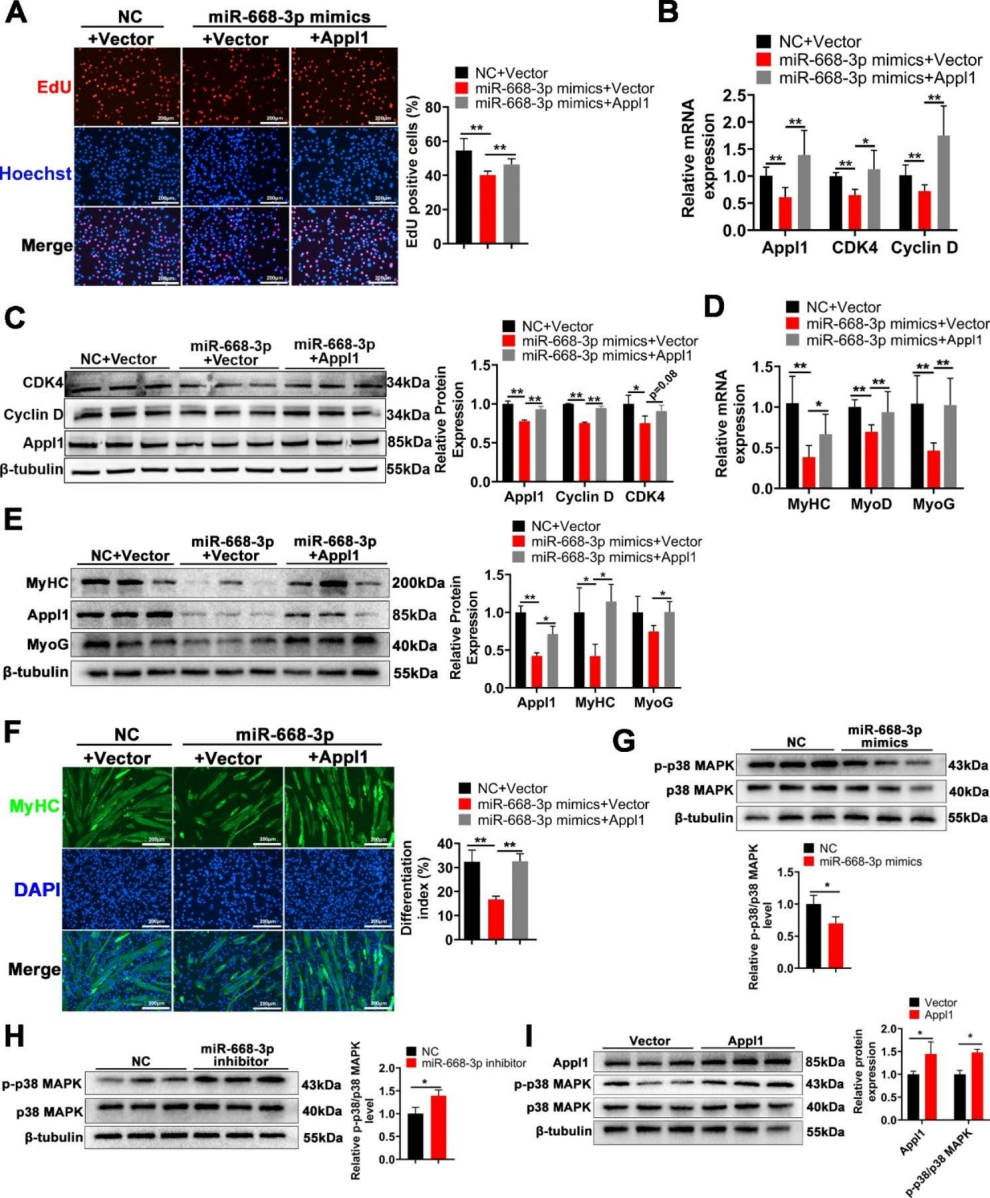
miR-668-3p inhibits myogenesis mainly by targeting Appl1 and inhibiting MAPK signalling. **A** EdU staining experiments were performed after cotransfection of miR-668-3p and Appl1. Quantification of the ratio of EdU-positive cells/total cells. **B** The mRNA expression levels of the proliferation genes CDK4 and Cyclin D after cotransfection of miR-668-3p and Appl1. **C** The protein expression levels of the proliferation genes CDK4 and Cyclin D after cotransfection of miR-668-3p and Appl1. The membrane was cleaved prior to hybridization with the antibody, and the protein bands in Figure C are from the same sample. Full-length blots/gels are presented in Supplementary Fig. S5A. **D** The mRNA expression levels of the differentiation genes MyHC, MyoD and MyoG after cotransfection of miR-668-3p and Appl1. **E** The protein expression levels of the differentiation genes MyHC and MyoG after cotransfection of miR-668-3p and Appl1. The membrane was cleaved prior to hybridization with the antibody, and the protein bands in Figure E are from the same membrane. Full-length blots/gels are presented in Supplementary Fig. S5B. **F** MyHC immunofluorescence staining and differentiation index after cotransfection of miR-668-3p and Appl1. **G** The levels of phosphorylated p38 MAPK after overexpression of miR-668-3p. The membrane was cleaved prior to hybridization with the antibody, and the protein bands in Figure G are from the same sample. Full-length blots/gels are presented in Supplementary Fig. S5C. **H** The levels of phosphorylated p38 MAPK after miR-668-3p knockdown. The membrane was cleaved prior to hybridization with the antibody, and the protein bands in Figure H are from the same sample. Full-length blots/gels are presented in Supplementary Fig. S5D. **I** The levels of phosphorylated p38 MAPK after overexpression of Appl1. The membrane was cleaved prior to hybridization with the antibody, and the protein bands in Figure I are from the same sample. Full-length blots/gels are presented in Supplementary Fig. S5E. The data represent the mean ± SD from at least three independent experiments. (*, *P* < 0.05; **, *P* < 0.01)

To demonstrate that miR-668-3p inhibits myogenesis mainly by targeting Appl1, we cotransfected miR-668-3p and Appl1 overexpression vectors into C2C12 cells. EdU staining showed that overexpression of Appl1 effectively attenuated the inhibitory effect of miR-668-3p mimics on myoblast proliferation (Fig. 6A). Additionally, RT‒qPCR showed that Appl1 reversed the inhibitory effect of miR-668-3p on *CDK4* and *Cyclin D* expression (Fig. 6B). Moreover, Appl1 significantly reversed the inhibitory effect of miR-668-3p on the expression of Cyclin D protein, and also reversed the expression of CDK4 protein to some extent. (Fig. 6C). Furthermore, we explored whether Appl1 mediates the role of miR-668-3p in the differentiation stage of myoblasts. Overexpression of Appl1 reversed the effects of miR-668-3p on repressing the expression of the differentiation genes MyoD, MyoG and MyHC (Fig. 6D, E). Similarly, immunofluorescence staining of MyHC revealed that overexpression of Appl1 alleviated the inhibitory effect of miR-668-3p on myogenic differentiation (Fig. 6F). In conclusion, these results suggest that miR-668-3p inhibits myogenesis mainly by targeting Appl1.

**Fig. 7:**
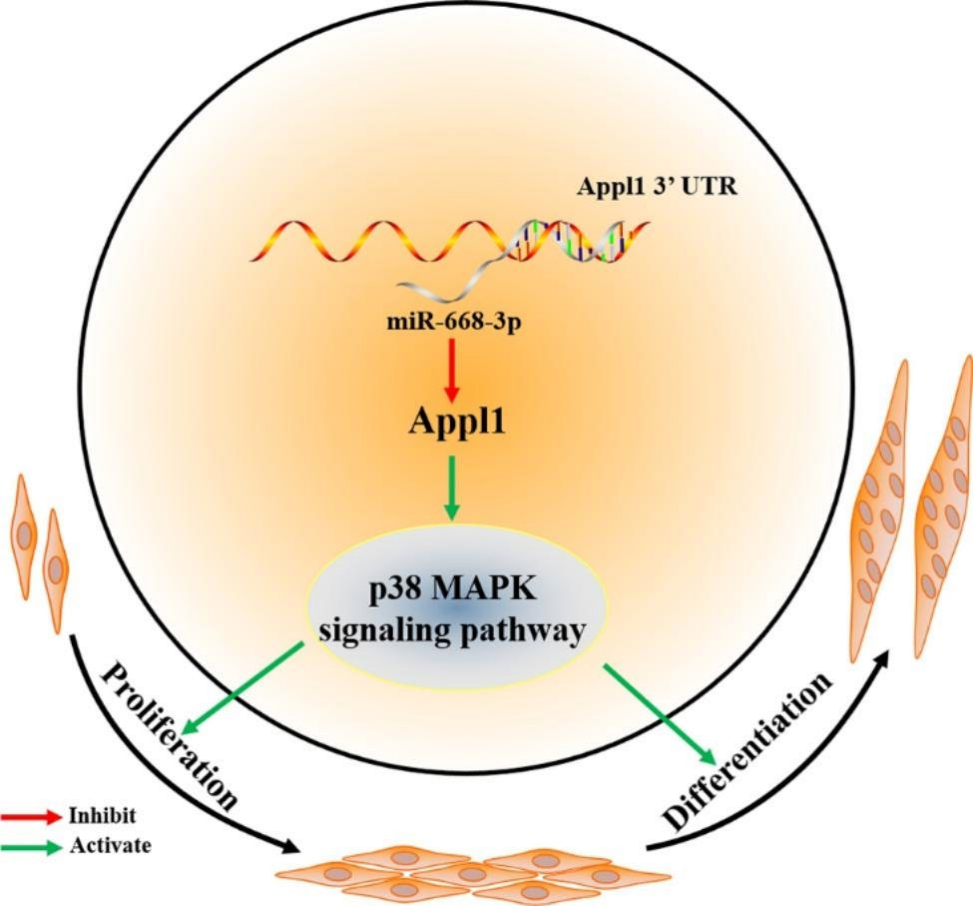
Molecular regulatory mechanism of miR-668-3p. miR-668-3p inhibits myoblast proliferation and differentiation by inhibiting the Appl1/p38 MAPK pathway

Appl1 can activate the p38 MAPK pathway [25]. p38 MAPK is involved in myogenesis [27]. Therefore, we speculated that miR-668-3p inhibits myogenesis by repressing the p38 MAPK signalling pathway by targeting Appl1. To reveal the potential mechanism by which miR-668-3p/Appl1 regulates myogenesis, we examined whether the p38 MAPK pathway was activated by miR-668-3p. After transfection of miR-668-3p mimics, we found that the level of phosphorylated p38 MAPK was decreased (Fig. 6G), while the level of phosphorylated p38 MAPK was increased after miR-668-3p inhibition (Fig. 6H). Moreover, after Appl1 overexpression, we found that the level of phosphorylated p38 MAPK was increased significantly (Fig. 6I). These results demonstrated that miR-668-3p inhibits myoblast proliferation and differentiation by inhibiting the Appl1/p38 MAPK pathway (Fig. 7).

## Discussion

Skeletal muscle development is a highly coordinated event that is dependent on a complex molecular regulatory network [3, 28–30]. In the past few decades, substantial evidence has been reported about the molecular network that regulates skeletal muscle growth and development, including noncoding RNAs [31, 32]. Increasingly, studies have shown that miRNAs are extensively involved in myogenesis and muscle diseases [33].

The existing research on miR-668-3p has mainly focused on its role in diseases. Wei et al. first reported that miR-668 was upregulated in a mouse model of renal ischaemia/reperfusion (I/R) injury and verified that miR-668 represses the mitochondrial fission protein MTP18 to prevent mitochondrial fragmentation and protect renal tubular cells from apoptosis [20, 34]. Subsequent studies on miR-668-3p have mainly focused on I/R injury. In this study, we found that miR-668-3p was highly expressed in skeletal muscle and differentially expressed during myogenic differentiation, while the role of miR-668-3p during myogenesis has not been reported. Therefore, it is necessary to have an in-depth understanding of the role of miR-668-3p in skeletal muscle.

To study the effects of miR-668-3p on the proliferation and differentiation of myoblast cells, miR-668-3p mimics and inhibitor were transfected into cells to verify their functions via both positive and negative mechanisms. We found that miR-668-3p inhibited the proliferation and differentiation of myoblasts. Furthermore, we predicted and demonstrated that Appl1 is a direct target gene of miR-668-3p that can promote the proliferation and differentiation of myoblasts. The phosphotyrosine binding domain of Appl1 binds to the cytoplasmic regions of adiponectin receptors 1 and 2(R1, R2) to regulate a series of adiponectin-induced signal transduction events, including the phosphorylation of AMPK and the phosphorylation of p38 MAPK [25]. We performed KEGG and GO analyses on the target genes of miR-668-3p, among which the MAPK signalling pathway attracted our attention. p38 MAPK is a major regulator of muscle development. p38 MAPK can activate muscle satellite cells and promote division and differentiation [35]. During the early stage of myoblast differentiation, p38 MAPK plays an active role in MRF transcription regulation and promotes myoblast fusion and muscle fibre formation [36]. Overexpression of Appl1 leads to the phosphorylation of p38 MAPK in myoblasts, activating the p38 MAPK signalling pathway. Therefore, we assume that miR-668-3p inhibits the p38 MAPK pathway by targeting Appl1. As expected, our results indicated that miR-668-3p/Appl1 regulates skeletal muscle myogenesis through the p38 MAPK pathway.

It is well known that skeletal muscle exert autocrine/paracrine effects, and adiponectin has also been proven to be produced and played by muscle cells [37]. As a key regulator of adiponectin in systemic energy homeostasis, whether Appl1 can also regulate myogenesis through other signalling pathways remains to be further verified. Similarly, Appl1/adiponectin plays a role in insulin regulation and glucose uptake. The type of muscle fibre in skeletal muscle is closely related to oxidative metabolism. Studies have shown that type IIA muscle fibres have higher adiponectin levels [38]. These higher adiponectin levels affect the oxidative capacity of skeletal muscle to some extent [39]. Therefore, it remains to be explored whether miR-668-3p/Appl1 has an impact on muscle fibre types, and its role in muscle diseases is also worthy of attention.

## Conclusions

In summary, our study suggests that miR-668-3p, which is a negative regulator of skeletal muscle development, can target the Appl1/p38 MAPK signalling pathway and inhibit myoblast proliferation and differentiation. These findings contribute to a new understanding of the mechanisms underlying skeletal muscle growth and development at the miRNA level and provide new potential insights into the regulation of skeletal muscle formation.

## Methods

### Animals and animal care

C57BL/6 male mice were purchased from the Fourth Military Medical University Animal Center (Xi’an, China). The animal surgeries were performed in compliance with the ARRIVE guidelines. All the animal care and sampling collection procedures were carried out in strict accordance with the protocol that was approved by the Institutional Animal Care and Institutional Ethics Committee of Northwest A&F University (NWAFU-314,020,038).

### Cell culture and transfection

Mouse C2C12 cells (ATCC, Rockefeller, New York, NY) and human embryonic kidney 293T cells were cultured in high glucose Dulbecco’s modified Eagle’s medium (DMEM, Servicebio, G4510) supplemented with 10% foetal bovine serum (ZETA LIFE, Z7186FBS-500) and 100 IU/mL penicillin‒streptomycin at 5% CO_2_ and 37 °C. Myoblast differentiation was induced with differentiation medium (DMEM supplemented with 2% horse serum, Solarbio, S9050) when the cells reached 90% confluence. The cells were collected at different time points. The culture medium was changed every other day. miR-668-3p mimics, NC, inhibitor, inhibitor NC (GenePharma, Shanghai, China) or pcDNA3.1, Appl1(General Biol, Anhui, China) were transfected into the cells using Lipofectamine™ 2000 (Invitrogen, 11,668,019). The CDS region of Appl1 was connected to the pcDNA3.1 vector, which was completed by General Biology Systems Ltd. For a six-well plate, add 2000 ng Appl1 to one well. For studies on proliferation, transfection was performed when cell confluence reached 50%, and for studies on differentiation, transfection was performed when cell confluence reached 80%. The medium was replaced with GM (Growth Medium) or DM (Differentiation Medium) at 4 to 6 h after transfection.

### RNA isolation and real-time qPCR

The total RNA was extracted from cells and tissues with AG RNAex Pro Reagent (Accurate Biology, AG21102) according to the manufacturer’s instructions. cDNA synthesis was performed with reverse transcription kits (Vazyme, R312-02 is used for cDNA synthesis of microRNA, and R323-01 is used for cDNA synthesis of other genes.) according to standard processes. Quantitative real-time PCR (RT‒qPCR) was performed using the StepOnePlus Real-time PCR system (Applied Biosystems, 2,720,500,631) with ChamQ SYBR qPCR Master Mix (Vazyme, Q311-02). The PCR protocol was as follows: denaturation at 95 ℃ for 30 s, followed by 40 cycles of 95 ℃ for 10 s, and 60 ℃ for 30 s. U6 was used as the internal reference gene for miR-668-3p, and GAPDH was used as the internal reference gene for other genes. The 2^−ΔΔCt^ method was used to analyzes RT‒qPCR data. U6 and miRNA primers were purchased from RiboBio (Guangzhou, China). The gene-specific primer sequences that were used in this study are listed in Table 1.

**Table 1:** The primer sequences for real-time qPCR

Genes	Primer sequences	Product length
Cyclin D	F: CAAAATGCCAGAGGCGGATG R: GGAGGGGGTCCTTGTTTAGC	272
PCNA	F: TTGCACGTATATGCCGAGACC R: GGTGAACAGGCTCATTCATCTCT	183
Cyclin E	F: CAGAGCAGCGAGCAGGAGC R: GCAGCTGCTTCCACACCACT	73
p21	F: AGTGTGCCGTTGTCTCTTCG R: ACACCAGAGTGCAAGACAGC	311
p27	F: AGATACGAGTGGCAGGAGGT R: ATGCCGGTCCTCAGAGTTTG	171
MyHC	F: CAAGTCATCGGTGTTTGTGG R: TGTCGTACTTGGGCGGGTTC	158
MyoD	F: GGACCCAGGAACTGGGATATG R: TCATAGAAGTCGTCTGCTGTCTC	110
MyoG	F: CAGTGAATGCAACTCCCACAG R: TGGACGTAAGGGAGTGCAGA	134
Appl1	F: AGGTCATAGATGAGCTTAGTTCTTG	274
	R: AAATAATGCATCATGGTCTGGTG	
GAPDH	F: TGCTGAGTATGTCGTGGAGTCT R: ATGCATTGCTGACAATCTTGAG	179

### Western blotting

Total protein was extracted from myoblasts using RIPA buffer (Beyotime Biotechnology, P0013B) and protease inhibitor mix (AbMole BioScience, M7528) after washing the cells with PBS three times. All the extraction processes were performed according to standard protocols. Twenty micrograms of protein was electrophoresed on 10% SDS polyacrylamide gels, and the protein was transferred to polyvinylidene fluoride membranes (Millipore, IPVH00010), Then, the membranes were blocked with 5% BSA at 4 °C for 2 h and incubated with antibodies (1:1000) against cyclin E (Santa, sc-377,100), cyclin D (Abways, CY5404), p27 (Santa, sc-1641), p21 (Abways, CY5088), CDK4 (Abways, CY5827), PCNA (Abways, CY1245), MyHC (R&D Systems, MAB4470), MyoG (Novus Biologiacals, NB100-56510), MyoD (Novus Biologiacals, NBP1-54153), Appl1 (Abways, CY8185), p38α MAPK (Abways, CY5262), Phospho-p38α MAPK (Abways, CY6390), β-Tubulin (Abways, AB0039), and GAPDH (Abways, AB0036) at 4 ℃ overnight. After being washed with Tris buffered saline with Tween (TBST), the membranes were incubated with HRP goat anti-mouse IgG or goat anti-rabbit IgG secondary antibodies (BOSTER, China).

### Flow cytometry

C2C12 cells were grown in six-well plates until they reached 30–50% confluence for transfection, and the cells were collected at a confluence of 70%. Prechilled 70% ethanol was used to fix the cell precipitates. The samples were analyzed by Servicebio Technology Co., Ltd. (Wuhan, China).

### EdU imaging assay

The Cell-Light TM EdU (5-ethynyl-2’-deoxyuridine) Apollo®567 In Vitro Imaging Kit was purchased from RiboBio (Guangzhou, China). Myoblasts at the normal growth stage were incubated with 50 µM EdU culture medium for 2 h, which was prepared according to the kit instructions. Then, the cells that were labelled with EdU were dyed in Apollo reaction solution, and the cell nuclei were stained with Hoechst for 10 min. Then, the cells were observed by using an inverted fluorescence microscope (OLYMPUS, CKX53), and the data were analysed with Image J.

### Immunofluorescence assay

C2C12 cells were differentiated for 2 or 4 days, fixed with 4% paraformaldehyde and blocked with 5% BSA for 30 min. The cells were then incubated overnight at 4 °C with MyHC primary antibody (1:200). Next, the samples were incubated with anti-mouse IgG (1:500) for 1 h at room temperature and DAPI for 10 min. Images were obtained from a Cell Imaging Microplate Detection System (Bio Tek, Cytation5). The differentiation index was determined by comparing the MyHC-positive cells to total nuclei.

### Luciferase reporter assays

The 3’-UTR of Appl1 was synthesized by General Biology Systems Ltd. (Anhui, China). Human embryonic kidney 293T cells (Stem Cell Bank, Chinese Academy of Science) were seeded at 8000 cells per well in a 48 well culture plate, and 250 ng of psiCHECK2-Appl1-3’-UTR was cotransfected with 50 nM of either miR-668-3p mimics or NC when the cells reached 70% confluence. After transfection for 48 h, the relative luciferase activities of Renilla compared with those of firefly were measured with a Dual-Luciferase reporter assay system (Promega, E1910) according to the manufacturer’s protocol.

### Bioinformatic analysis

The miRNA sequences were searched at miRBase (http://www.miRbase.org/), and the 3’-UTR sequences of Appl1 were downloaded from NCBI. Target genes of miRNA were predicted by TargetScan 7.1 mouse (http://www.targetscan.org), miRDB (http://www.miRdb.org/miRDB/) and miRWalk (http://mirwalk.umm.uni-heidel berg.de/). The data of GO term analysis and KEGG (Kyoto Encyclopedia of Genes and Genomes) pathway analysis comes from ENCORI (https://starbase.sysu.edu.cn/index.php) [40–43].

### Statistical analysis

All the graphs were generated using GraphPad Prism 8.02. The data are expressed as the means ± SDs. The significance of the differences between groups was assessed using Student’s t test or one-way or two-way analysis (*, *P* < 0.05; **, *P* < 0.01).

## Electronic supplementary material

Below is the link to the electronic supplementary material.

Supplementary Material 1

## Data Availability

The raw data that supports the findings of this study are available from the corresponding author upon reasonable request.

## Abbreviations

Appl1: Adaptor protein, phosphotyrosine interacting with PH domain and leucine zipper 1
miRNA: microRNA
Sol: Soleus
EDL: Extensor digitorum longus
PCNA: Proliferating cell nuclear antigen
CDK4: Cyclin dependent kinase 4
P21: Cyclin dependent kinase inhibitor 1a
P27: Cyclin dependent kinase inhibitor 1b
MyoD: Myogenic differentiation 1
MyoG: Myogenin
MyHC: Myosin heavy chain
EdU: 5-ethynyl-2’-deoxyuridine
DAPI: 4’,6-diamidino-2-phenylindole
GAPDH: Glyceraldehyde-3-phosphate dehydrogenase
NC: Negative control
GO: Gene Ontology
KEGG: Kyoto Encyclopedia of Genes and Genomes
DMEM: Dulbecco’s modified Eagle’s medium
RT-qPCR: Real-time quantitative PCR.
