# Supplementary material for: MicroRNA-668-3p inhibits myoblast proliferation and differentiation by targeting Appl1
Source: BMC Genomics. 2023 Jul 24;24:415. doi: 10.1186/s12864-023-09431-0 (PMC10364376; doi:10.1186/s12864-023-09431-0)
Supplement: Supplementary file 1 — Supplementary Material 1 [file 12864_2023_9431_MOESM1_ESM.docx]

**MicroRNA-668-3p inhibits myoblast proliferation and differentiation by targeting Appl1**

Haigang Cao^1†^, Tianning Du^1, 2†^, Chenchen Li^1^, Lingling Wu^1^, Jieming Liu^1^, Yuan Guo^1^, Xiao Li^1^, Gongshe Yang^1^, Jianjun Jin^1*^and Xin’e Shi^1*^

^1^Laboratory of Animal Fat Deposition and Muscle Development, Key Laboratory of Animal Genetics, Breeding and Reproduction of Shaanxi Province, College of Animal Science and Technology, Northwest A&F University, Yangling, Shaanxi, China.

^2^Microbial Research Institute of Liaoning Province, Chaoyang, Liaoning, China.

^†^There authors contributed equally to this work.

*For correspondence: Xin’e Shi: xineshi@nwafu.edu.cn; Jianjun Jin: jinjianjun2021@nwafu.edu.cn.

**
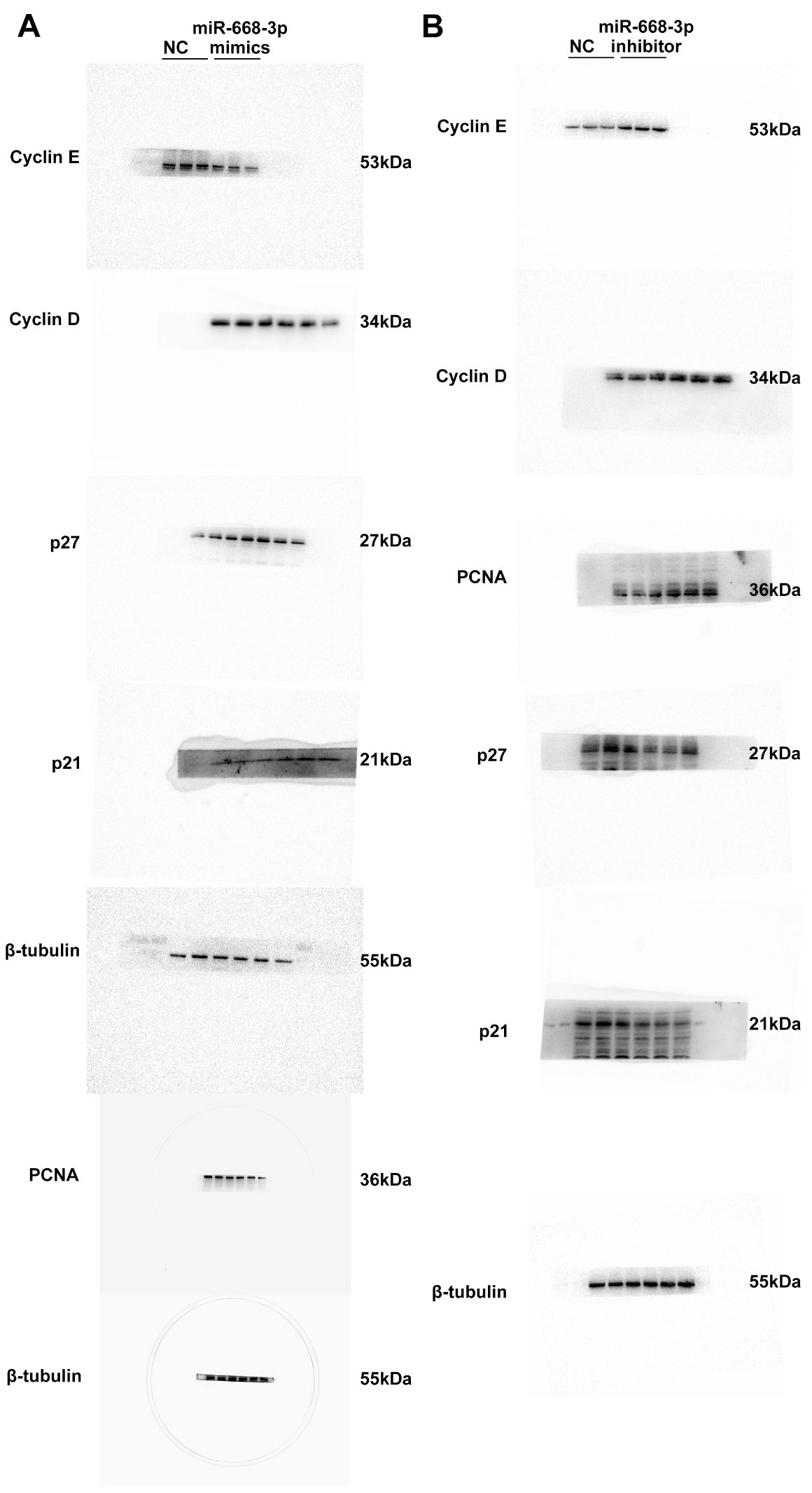
**

**Fig. S1** miR-668-3p inhibits myoblast proliferation. **A** The full-length blots/gels of Cyclin E, Cyclin D, p27, p21 and PCNA proteins after overexpression of miR-668-3p. **B** The full-length blots/gels of Cyclin E, Cyclin D, PCNA, p27 and p21 proteins after inhibition of miR-668-3p.

**
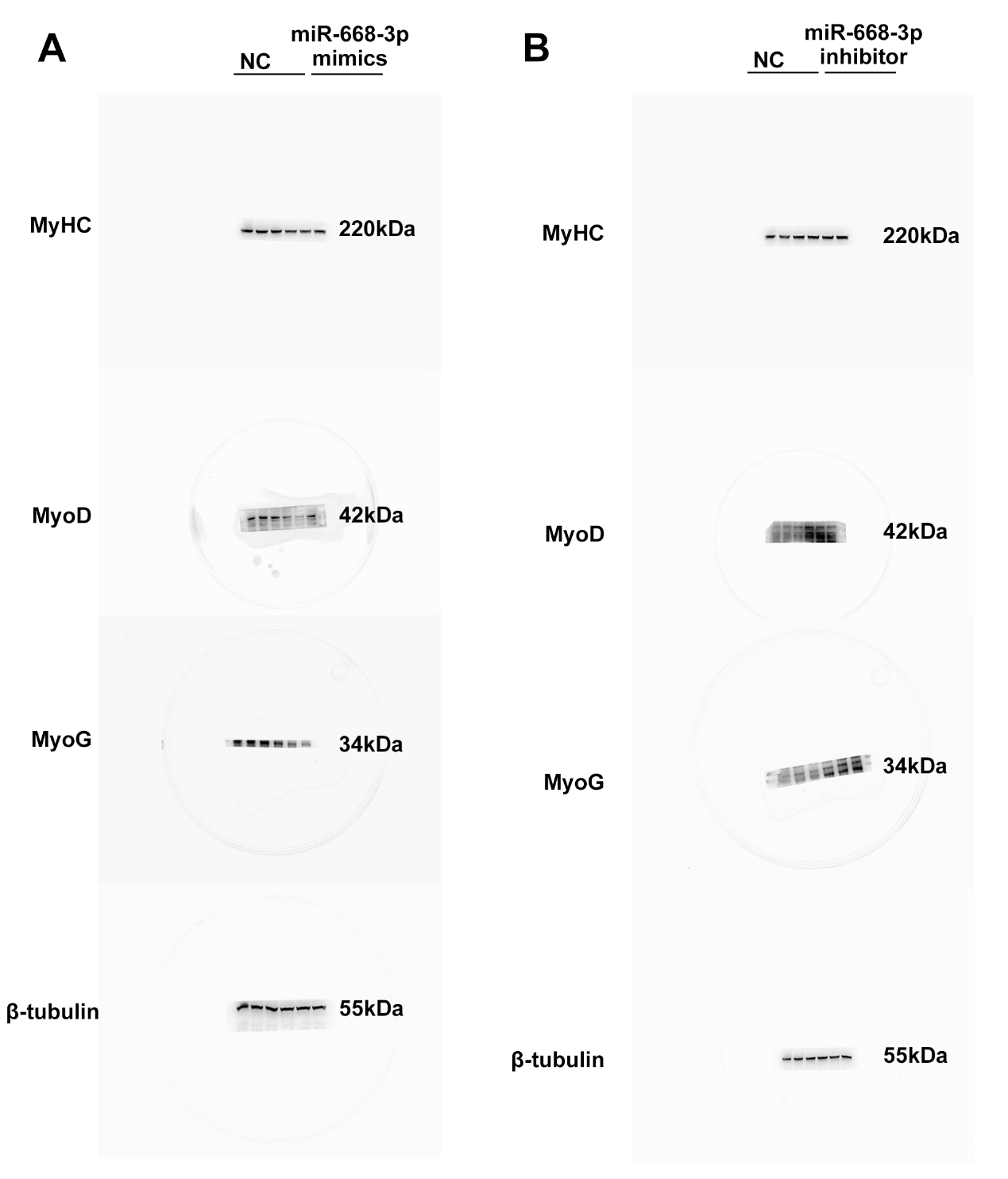
**

**Fig. S2** miR-668-3p inhibits myogenic differentiation. **A, B** The full-length blots/gels of MyHC, MyoD, and MyoG proteins after overexpression or inhibition of miR-668-3p.

**
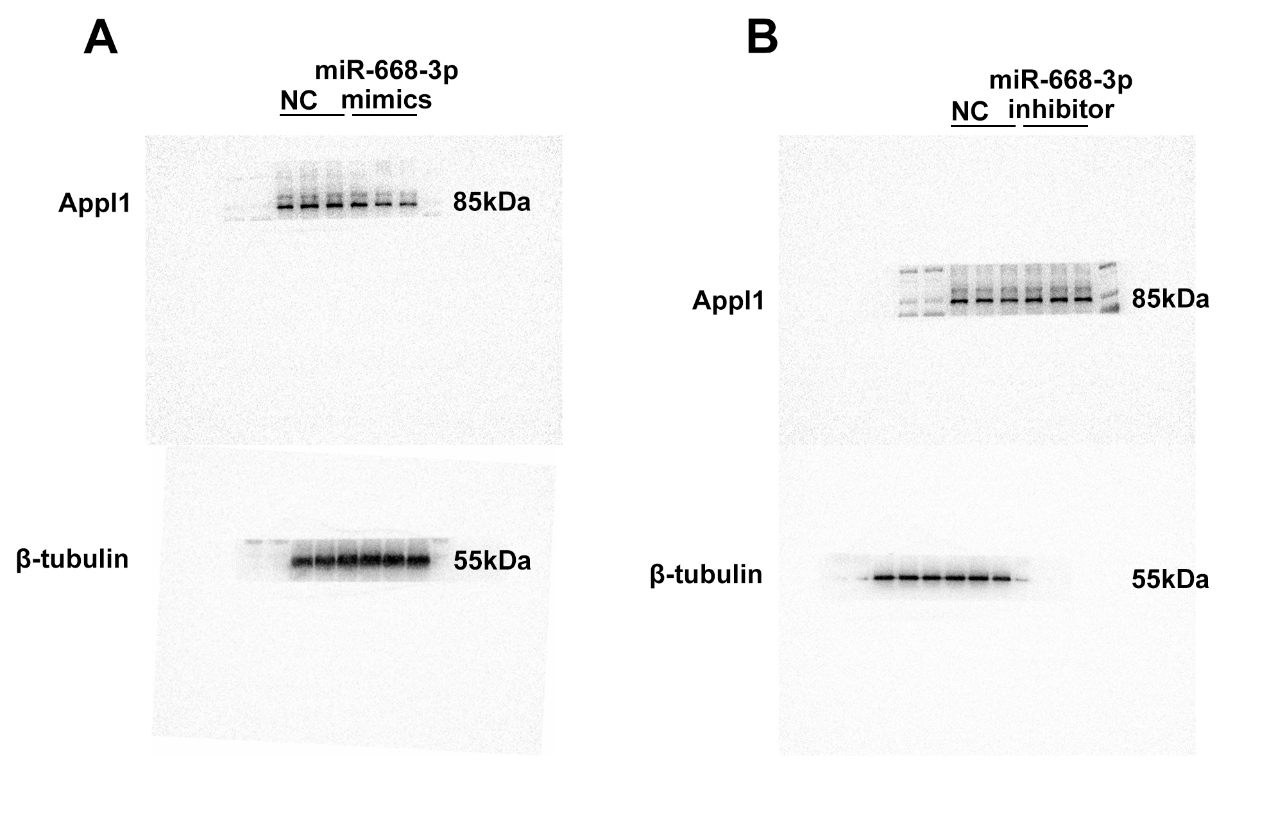
**

**Fig. S3** miR-668-3p directly targets Appl1. **A, B** The full-length blots/gels of Appl1 protein after overexpression or inhibition of miR-668-3p.

**
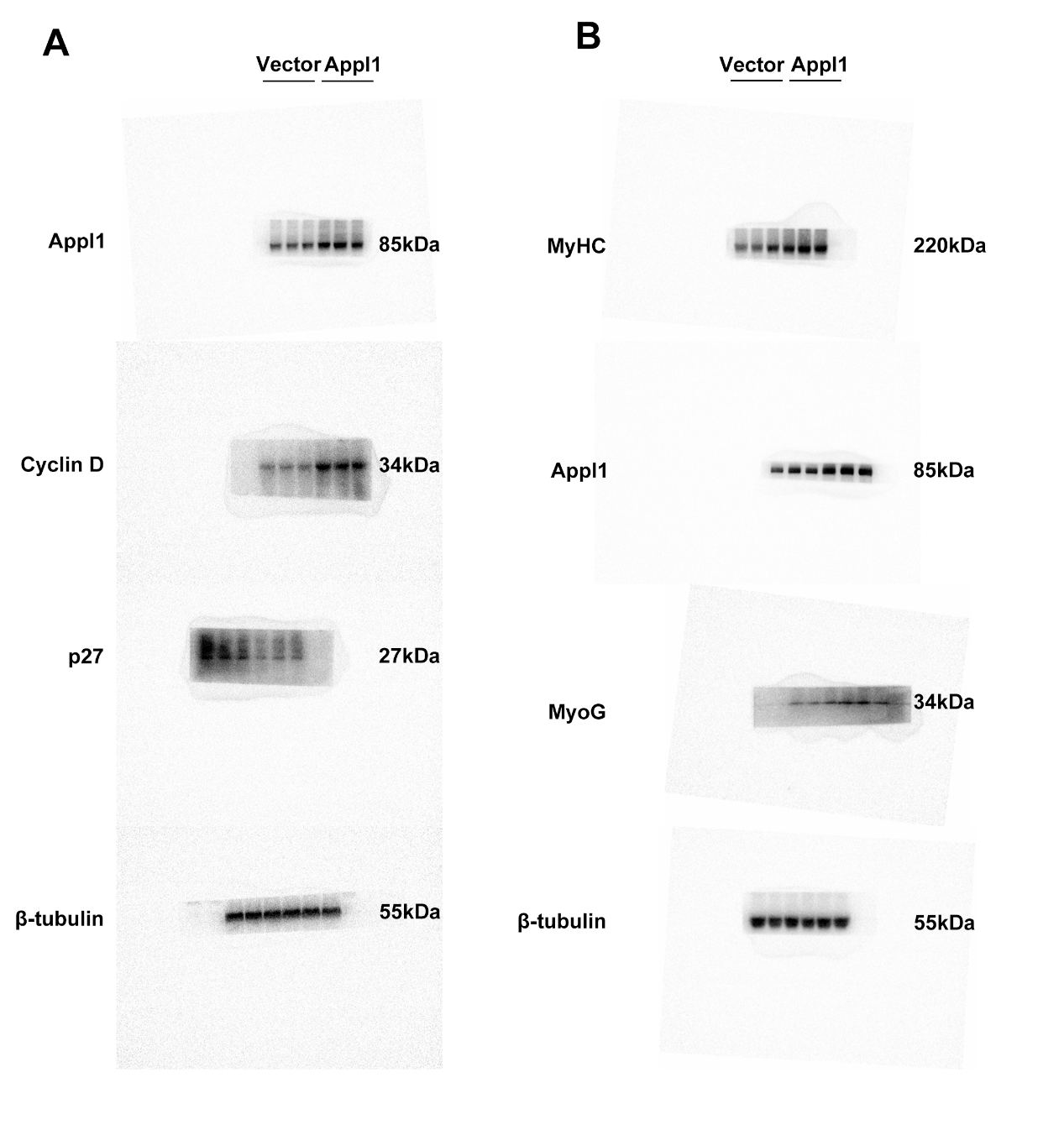
**

**Fig. S4** Appl1 promotes myoblasts proliferation and differentiation. **A** The full-length blots/gels of Appl1, Cyclin D, and p27 proteins after overexpression of Appl1. **B** The full-length blots/gels of Appl1, MyHC and MyoG proteins after overexpression of Appl1.

**
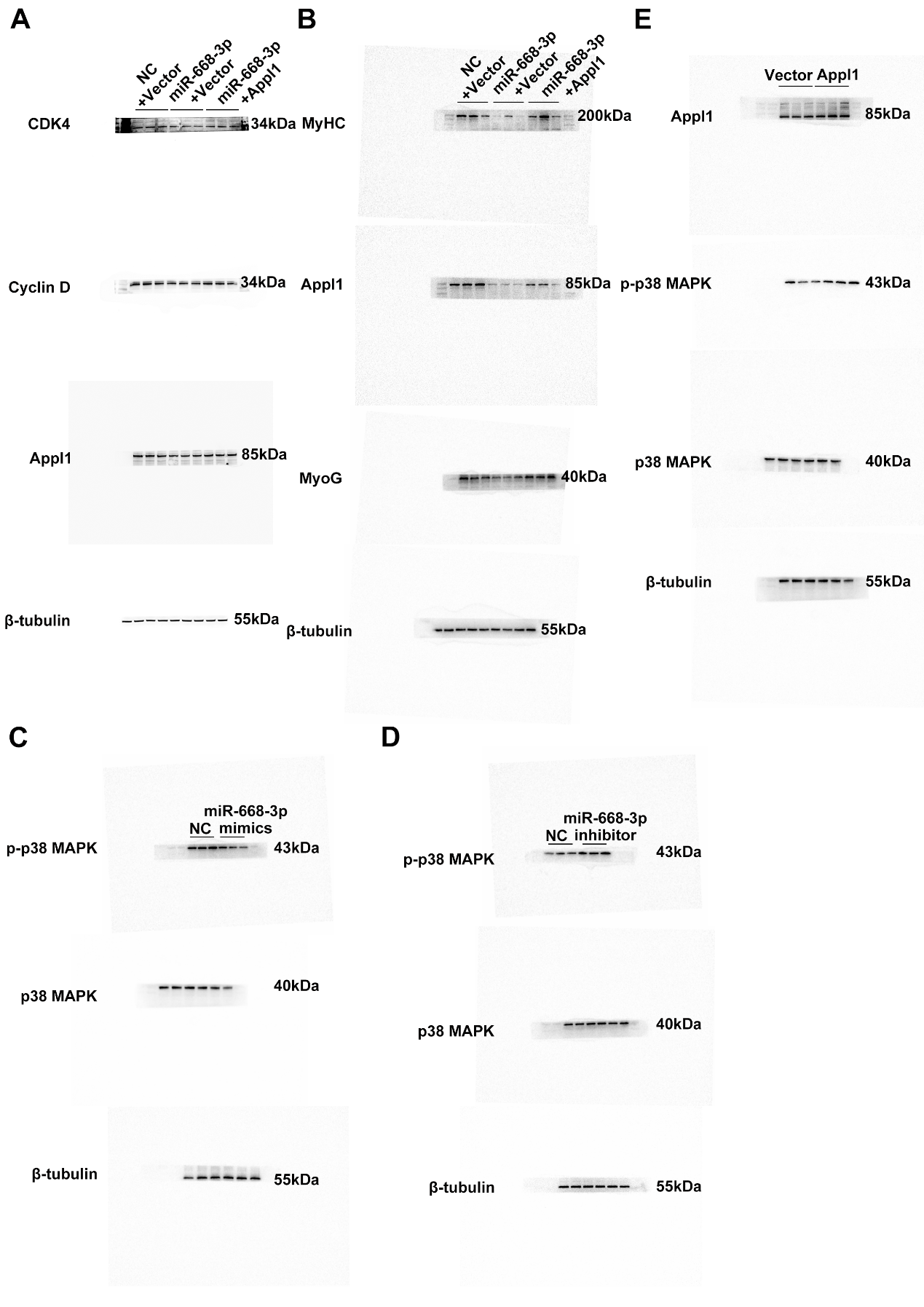
**

**Fig. S5** miR-668-3p inhibits myogenesis mainly by targeting Appl1 and inhibiting MAPK signaling. **A** The full-length blots/gels of Appl1, Cyclin D, and CDK4 proteins after co-transfection of miR-668-3p and Appl1. **B** The full-length blots/gels of Appl1, MyHC and MyoG proteins after co-transfection of miR-668-3p and Appl1. **C, D** The full-length blots/gels of p38 MAPK and p-p38 MAPK proteins after overexpression or inhibition of miR-668-3p. **E** The full-length blots/gels of Appl1, p38 MAPK and p-p38 MAPK proteins after overexpression of Appl1.
